# A Case of Malignant Melanoma Arising in Mediastinal Malignant Teratoma

**DOI:** 10.1155/2018/1306824

**Published:** 2018-12-31

**Authors:** Ikuma Nozaki, Yumi Tone, Junko Yamanaka, Hideko Uryu, Yuko Shimizu-Motohashi, Noriko Sato, Takeji Matsushita

**Affiliations:** ^1^National Center for Global Health and Medicine, Tokyo, Japan; ^2^Waseda Takenoko Clinic for Children, Tokyo, Japan

## Abstract

We report about a 14-year-old boy who presented with an anterior mediastinal mass that was diagnosed as malignant teratoma. Surgical resection was performed along with pre- and postoperative chemotherapy. Although elevated alpha-fetoprotein became negative, he experienced pain in his right hip joint 3 months after resection. Systematic evaluation revealed multiple locations of metastasis, and the pathological diagnosis based on bone biopsy was malignant melanoma originating from malignant teratoma, which rapidly progressed. He died 15 months after diagnosis of the original malignant teratoma. Diagnosing and treating malignant transformation of teratoma, including malignant melanoma, is difficult because it is very rare. To our knowledge, this is the second reported case of malignant melanoma arising from a mediastinum malignant teratoma, with both cases having a poor prognosis. In addition to the follow-up of tumor markers, systematic evaluation, including imaging, should be considered even after remission to monitor malignant transformation of teratoma. We expect to establish a successful therapy and improve mortality rate after more such cases are accumulated.

## 1. Introduction

Primary mediastinal germ cell tumors (GCTs) are rare and account for approximately 5% of all extragonadal GCTs. In addition, they account for approximately 25% of mediastinal tumors in children, including 60% of mature teratoma, 20% of mixed GCTs, and 20% of embryonal carcinoma (including seminoma/germinoma, immature teratoma, yolk sac tumor, and choriocarcinoma) [1, 2]. The prognosis of mediastinal GCTs is poor compared to that of gonadal nonseminomatous GCTs (NSGCTs) [3, 4] because the 5-year overall survival rate of mediastinal GCTs is much lower than that of gonadal NSGCTs. Recently, survival rates of pediatric GCTs have significantly improved because of a combination of tumorectomy and because 80% of patients receive platinum-based chemotherapy [3].

However, here, we report about a patient with mediastinum malignant teratoma who could not be saved despite surgical resection and pre- and postoperative chemotherapy because of the metastasis of malignant melanoma arising from a teratoma.

## 2. Case Presentation

**Figure 1:**
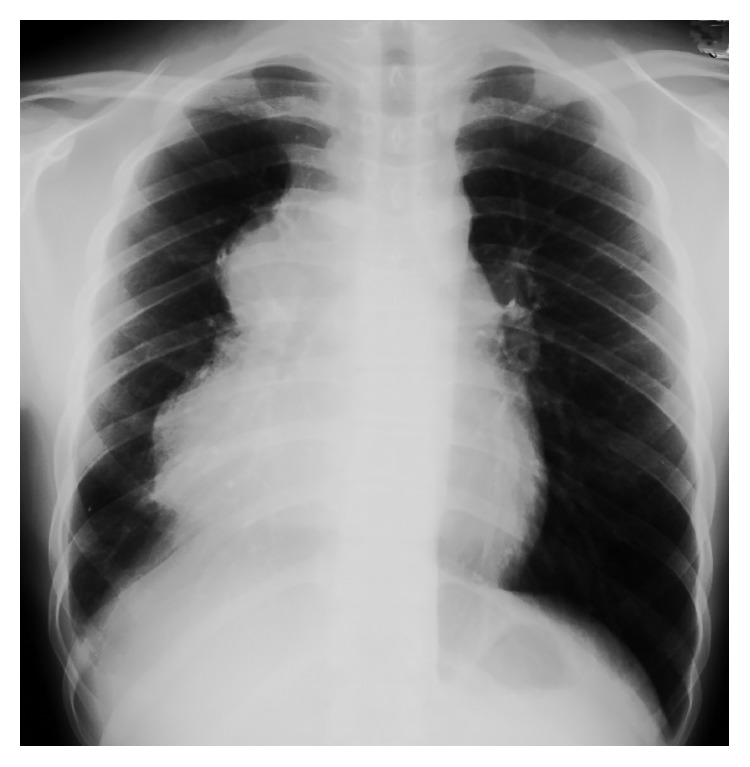
Chest X-ray showing an anterior mediastinum mass.

**Figure 2:**
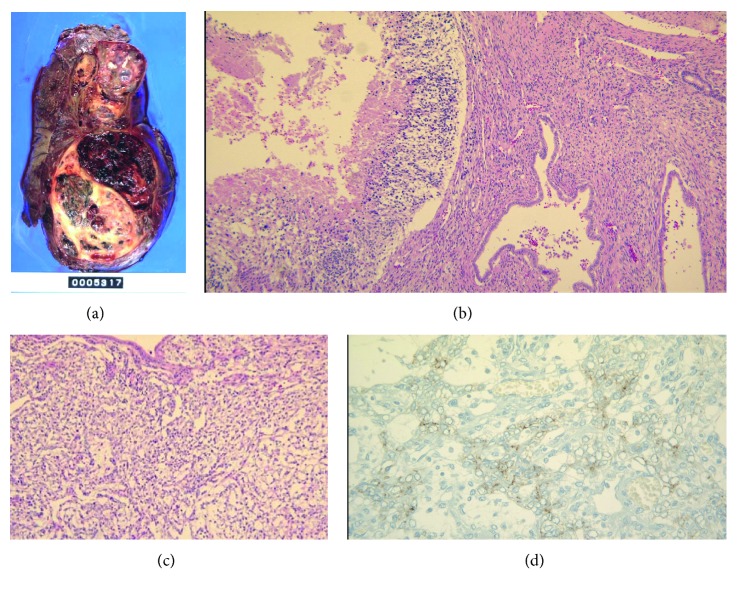
Pathology of the extracted tumor: the image shows the mediastinal tumor resection tissue (19 × 13 × 11.5 cm in size). The surface is covered with a membrane, and the boundary is clear and shows expansive development. Approximately two-thirds of the cut surface show hemorrhaging and necrosis, including the cystic area of grayish white or black color. The remaining tumor tissue consists of a teratoma showing epithelial/epidermal appendages, central nerve, bone/cartilage, smooth muscle, adipose tissue, gastrointestinal epithelium, and respiratory epithelium differentiation. Mesenchymal tissue, muscle/adipose tissue, cartilage, central nervous system, and ducts can be seen as undifferentiated immature components. Several yolk sac tumor lesions showing reticular structures with positive immunostaining for alpha fetoprotein can also be seen.

A 14-year-old boy, who was initially examined for continuous coughing, was referred to our hospital owing to an anterior mediastinal mass identified on chest X-ray (Figure 1). Systematic examination revealed a 20 × 10 cm sized mass at the right anterior mediastinum that involved the right pulmonary vein and elevated levels of alpha fetoprotein (AFP), a tumor marker, at 3825 ng/ml. Needle aspiration was performed, but only necrotic tissue could be collected. We did not analyze the karyotype since the symptoms suggesting the Klinefelter syndrome were not observed. Therefore, a clinical diagnosis of malignant teratoma was made, and three courses of cisplatin-based chemotherapy were administered because AFP levels continuously increased. After chemotherapy, AFP levels decreased, although the size of the tumor did not change, as evident in a computed tomography (CT) scan. Next, tumorectomy and total right lung extraction were performed. The pathological diagnosis of the extracted tumor was malignant teratoma with areas of yolk sac tumor (Figure 2). No cancer cells were found at the edges of the area where the tumor was removed. Two courses of cisplatin-based postoperative chemotherapy were administered until AFP levels were normal.

**Figure 3:**
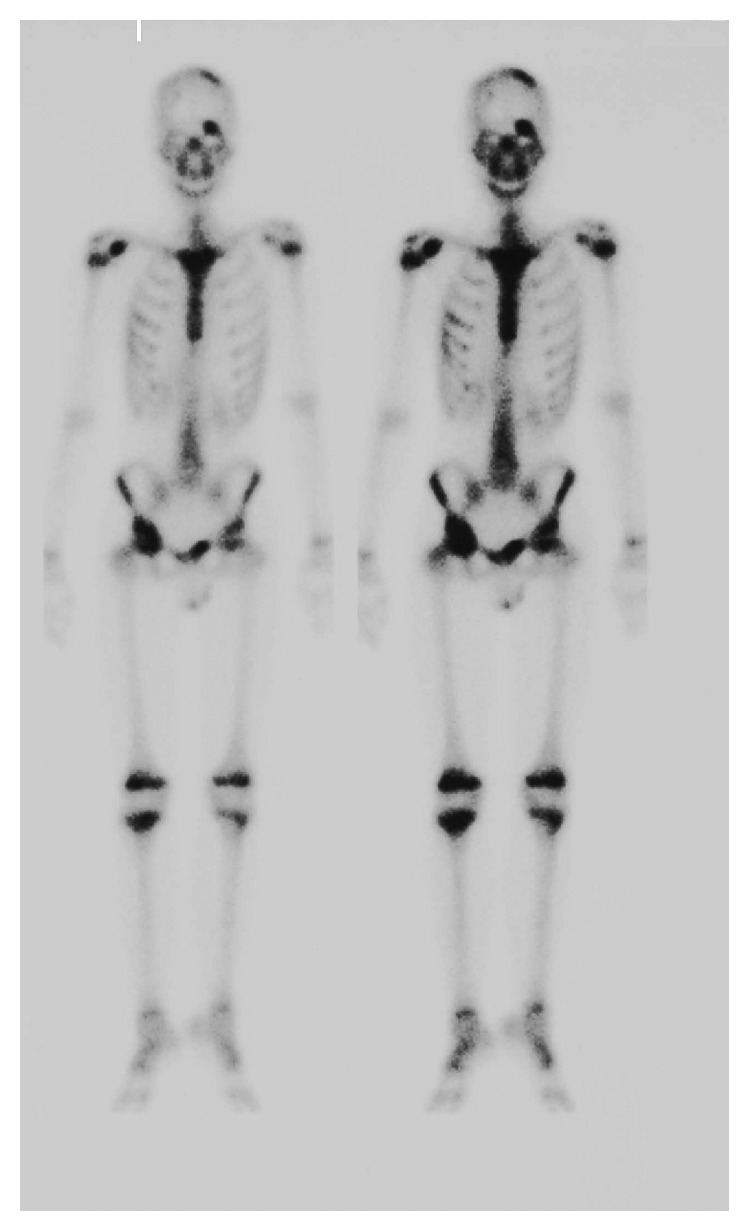
Bone scintigraphy showing multiple lesions in the left frontal bone, sternum, right superior supraclavicular fossa, and iliac bone, suggesting fractures, inflammation, or malignant cells.

**Figure 4:**
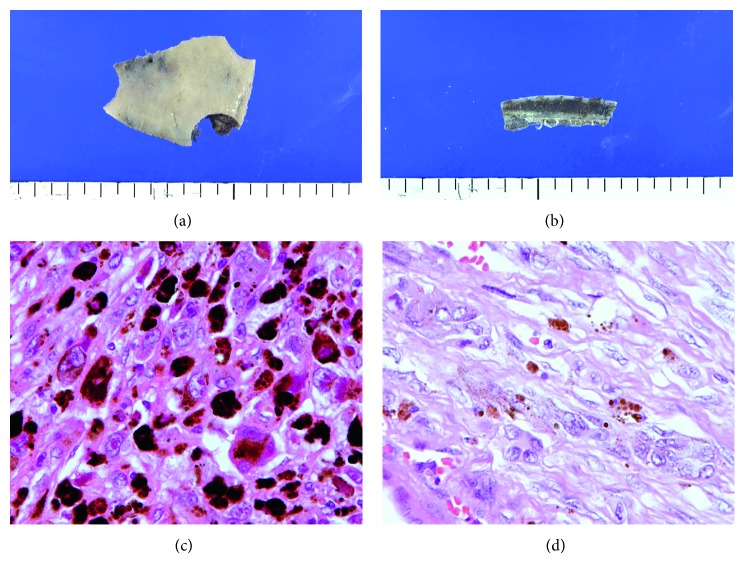
Cranial bone biopsy: black lesions are observed macroscopically at the surface and in the lumen of specimen. Histologically, metastasis of large heterozygous malignant cells is observed in the medullary cavity. Tumor cells are round or polygonal, including multinucleated cells with clear nucleoli, which have melanin granules in a wide cytoplasm. The histology is consistent with malignant melanoma. Large heteromorphic cells containing melanin were found with mesenchymal spindle-shaped cells of primary immature mediastinum teratoma.

After 3 months of follow-up, the patient experienced pain in his right hip joint while AFP was still normal. The ^99m^Tc-methylene diphosphonate bone scan showed increased tracer uptake at the left forehead and right hip joint (Figure 3). Head and pelvic MRI also revealed signs of metastasis at the left forehead and right hip joint. Abdominal CT showed a metastatic region in the liver. Bone biopsy was performed at the left forehead. The pathological diagnosis was metastatic malignant melanoma originating from an immature teratoma of mediastinum (Figure 4). Large heteromorphic cells with melanin were found in the original mediastinal malignant teratoma by retrospective re-examination. This suggested that a section of the malignant melanoma in the original malignant teratoma, which was composed of various components, metastasized. Because of the very fast disease progression, after consultation with the family, aggressive treatment was discontinued, and palliative therapy was provided. He died 15 months after diagnosis of the original malignant teratoma.

## 3. Discussion

We report about an adolescent patient who died of metastatic malignant melanoma arising from a mediastinal malignant teratoma after two courses of chemotherapy without re-elevation in tumor markers, including AFP. There are few reported cases of malignant melanoma arising from ovarian cystic teratomas [2, 5–10], with Algeri et al. reporting 49 cases in their review article. However, to our knowledge, this is only the second reported case of melanoma arising from a malignant mixed germ cell tumor of the anterior mediastinum [11]. Thus, clinical manifestations remain unclear because of its rarity.

A review of cases of primary malignant melanoma of the ovary revealed that it is extremely rare and has poor prognosis [9]. The strongest prognostic factors reported in that review article were negative resection margins and stage at diagnosis. In addition, serum levels of tumor markers, such as AFP and HCG, are well-known indicators used for diagnosis and follow-up of mediastinal GCTs [12]. In our case, serum AFP levels were high at the time of diagnosis and decreased after surgical resection and series of chemotherapies. The pathological examination of a surgical specimen also suggested complete resection was successful. However, the disease rapidly progressed without elevation of AFP levels and resulted in multiple metastases at the left forehead, right hip joint, and liver, which were discovered at the time of melanoma diagnosis, which indicates stage IV disease [13].

In a review of 9 cases in which malignant melanoma originated from ovarian cystic teratomas, patients were aged 24–75 years, with an average age of 51.1 years [7]. Distant metastases were found in four of nine patients, all of whom died owing to the disease at the time of publication. Two of five patients without metastasis were reported with no evidence of disease (NED), and follow-up of the other two was unknown. In the two cases of malignant melanoma that originated from mediastinal teratomas, including our case, both were young males aged 14 and 32 years. Distant metastasis was observed in our case, but there was no evidence of distant metastasis in the other case. Radiation therapy continued at the time of reporting for the other case. Screening of distant metastasis, including PET imaging, is strongly recommended [7]. Recently, options for effective treatment of malignant melanoma have been increasing; thus, detection of melanomatous malignant transformation should be considered for patients with mediastinum GCTs.

Mediastinal NSGCTs are associated with hematologic malignancies such as acute megakaryoblastic and myelogenous leukemia, myelodysplasia, malignant mastocytosis, and malignant histiocytosis. A retrospective study at 11 centers in the United States and Europe found that 6% of 287 patients with primary mediastinal NSGCTs developed a hematologic disorder [14]. Those cases had poor prognosis, the median survival following diagnosis was 5 months, and no patients survived >2 years.

Malignant transformation of teratomas, especially malignant melanomas, is very rare, and its diagnosis and treatment are extremely difficult. Although the data are limited, the prognosis is poor, and the overall 5-year mortality rate is estimated at 90% [7]. Prognosis of the disease is unpredictable even with various approaches, including combinations of surgery, chemotherapy, and/or immunotherapy, used in recently reported cases. In addition to the follow-up of tumor markers, systematic evaluation, including imaging, should be considered even after remission, to monitor the malignant transformation of teratoma. We expect to establish a successful therapy and improve the mortality rate after more such cases are accumulated.
